# Identification of A Risk Signature Based on Lactic Acid Metabolism-Related LncRNAs in Patients With Esophageal Squamous Cell Carcinoma

**DOI:** 10.3389/fcell.2022.845293

**Published:** 2022-05-12

**Authors:** Fangchao Zhao, Yishuai Li, Zefang Dong, Dengfeng Zhang, Pengfei Guo, Zhirong Li, Shujun Li

**Affiliations:** ^1^ Department of Thoracic Surgery, The Second Hospital of Hebei Medical University, Shijiazhuang, China; ^2^ Department of Thoracic Surgery, Hebei Chest Hospital, Shijiazhuang, China; ^3^ Clinical Laboratory Center, The Second Hospital of Hebei Medical University, Shijiazhuang, China

**Keywords:** esophageal squamous cell carcinoma, lactate metabolism, prognostic long noncoding RNA, prognostic model, immune microenvironment, chemotherapy response

## Abstract

Lactic acid, formerly thought of as a byproduct of glycolysis or a metabolic waste produced, has now been identified as a key regulator of cancer growth, maintenance, and progression. However, the results of investigations on lactic acid metabolism-related long non-coding RNAs (LRLs) in esophageal squamous cell carcinoma (ESCC) remain inconclusive. In this study, univariate Cox regression analysis was carried out in the TCGA cohort, and 9 lncRNAs were shown to be significantly associated with prognosis. Least absolute shrinkage and selection operator (LASSO) regression analysis and multivariate Cox regression analysis were then used in the GEO cohort. 6 LRLs were identified as independent prognostic factors for ESCC patients used to construct a prognostic risk-related signature subsequently. Two groups were formed based on the middle value of risk scores: a low-risk group and a high-risk group. Following that, we conducted Kaplan-Meier survival analysis, which revealed that the high-risk group had a lower survival probability than the low-risk group in both GEO and TCGA cohorts. On multivariate Cox regression analysis, the prognostic signature was shown to be independent prognostic factor, and it was found to be a better predictor of the prognosis of ESCC patients than the currently widely used grading and staging approaches. The established nomogram can be conveniently applied in the clinic to predict the 1-, 3-, and 5- year survival rates of patients. There was a significant link found between the 6 LRLs-based prognostic signature and immune-cell infiltration, tumor microenvironment (TME), tumor somatic mutational status, and chemotherapeutic treatment sensitivity in the study population. Finally, we used GTEx RNA-seq data and qRT-PCR experiments to verify the expression levels of 6 LRLs. In conclusion, we constructed a prognostic signature which could predict the prognosis and immunotherapy response of ESCC patients.

## Introduction

Esophageal cancer is the fifth most prevalent malignancy in China and the fourth leading cause of cancer-related fatalities (Anandavadivelan and Lagergren, 2016). Esophageal cancer may be classified into two subgroups based on its histological characteristics: esophageal adenocarcinoma (EAC) and esophageal squamous cell carcinoma (ESCC) (Smyth et al., 2017). ESCC accounts for nearly 90 percent of all esophageal cancer occurrences in China and is the most frequent histologic form of the disease (He et al., 2020). Despite the breakthroughs in the diagnosis, prognosis, and treatment of ESCC, early diagnosis still remains poor, with a 5-years overall survival rate less than 20% in some cases (Yang et al., 2020). The occurrence and development of ESCC relies on multiple factors, stages, and genes. Accordingly, both genetic and environmental factors affect the development of this disease (Abnet et al., 2018). Increasing our understanding of the molecular mechanisms underlying the development of ESCC and improving the efficiency of screening clinical indicators are crucial for early diagnosis and improved prognosis in patients with ESCC.

Metabolic reprogramming is an important feature of tumors, which is closely related to the occurrence, development and high invasiveness of tumors (Yoshida, 2015). Aerobic glycolysis is one of the important signs of tumor metabolic reprogramming. In an aerobic environment, tumor cells still choose glycolysis as the main source of energy, and a large amount of lactic acid is produced in this process (Abdel-Wahab et al., 2019; Abbaszadeh et al., 2020). Lactic acid is no longer regarded as a metabolic waste produced by glycolysis, it acts as an important signal molecule that affects the biological characteristics of surrounding cells (Hirschhaeuser et al., 2011). Lactic acid can also be used as an “alternative fuel” to establish metabolic coupling between cancer cells and adjacent cells to maintain tumor growth. At the same time, lactic acid can increase the malignancy of tumors by regulating the occurrence and development of tumors (Faubert et al., 2017; Pereira-Nunes et al., 2020).

Long non-coding RNAs (lncRNAs) are a class of transcripts longer than 200 nucleotides without protein-coding function (Zhou et al., 2016a; Zhao and Fan, 2019). LncRNAs emerge as a significant regulator responsible for various biological processes by regulating the expression of target genes from transcriptional, post-transcriptional and epigenetic levels (Zhou et al., 2016b; Zheng et al., 2021). In the research field of ESCC, lncRNAs have attracted wide concern and some abnormally expressed lncRNAs are considered as potential biomarkers or therapeutic targets for ESCC (Li et al., 2019). Increasing evidences have pointed out that cancer-related lncRNAs play an important role in metabolic reprogramming and promoting malignant phenotype (Xiao et al., 2016). For example, patients with hepatocellular carcinoma may benefit from a glycolysis-related lncRNA signature (Bai et al., 2021), and patients with colorectal cancer can benefit from fatty acid metabolism-related lncRNAs (Peng et al., 2021), which are possible indicators for predicting the overall survival. However, it remains unclear whether lactic acid metabolism-related lncRNAs (LRLs) are implicated in the development of ESCC.

In this study, a prognostic signature based on 6 LRLs was established and verified for the first time. Moreover, a significant correlation was observed between the lncRNA-based prognostic signature and immune-cell infiltration, tumor microenvironment (TME), tumor somatic mutations, and chemotherapeutic drug sensitivity. Therefore, our findings suggest that 6-LRL signature might be beneficial to the development of individualized treatments and improve the overall survival (OS) of patients with ESCC.

## Materials and Methods

### Datasets and Data Pre-Processing

The RNA-seq transcriptome data in fragment per kilobase method (FPKM) format and corresponding clinical data of ESCC patients were extracted from TCGA (ESCC tissue samples: 80, normal epithelial tissue samples: 11) and GEO database (GSE53624, ESCC tissue samples: 119). Subsequently, these data were collated and annotated, and then collapsed into protein-coding genes and lncRNAs employing the annotation documents from the GENCODE database. And 13,832 lncRNAs were identified. In addition, 262 lactic acid metabolism-related genes (LRGs) were extracted from MsigDB. FPKM data were converted to transcripts per kilobase million (TPM) data, and. batch effects between these cohorts were removed using “sva” package. Meanwhile, we used the “limma” package to explore the differential expression LRGs between normal samples and tumor samples (|Log2FC| > 1 and *p* < 0.05). Subsequently, Pearson correlation analysis was conducted using the 13,832 lncRNAs and differential expression LRGs identified (*p* < 0.001, correlation coefficient >0.6). Ultimately, 216 lactic acid metabolism-related lncRNAs (LRLs) were screened for follow-up bioinformatics analysis.

### Construction of A Risk Signature

In TCGA-ESCC chort, prognostic lncRNAs (*p*-value < 0.05) were screened using univariate Cox regression analysis. In GEO cohort, these prognostic lncRNAs were further incorporated into multivariate Cox and Least absolute shrinkage and selection operator (LASSO) regression analysis to identify lncRNAs involved in signature construction. We used the appropriate λ to build the signature and to control the complexity of LASSO regression. The risk score calculating formula is:

$$\sum_{i=1}^{n}Coef_{i}*x_{i}$$

The Coefi and xi represent the coefficient and expression levels of the corresponding lncRNA, respectively.

### Exploring Clinical Benefit

The risk score for every ESCC patient by summing according to the above formula. Risk signatures for predicting survival were assessed by the area under the curve (AUC) and Kaplan-Meier survival analysis. We calculated the risk score of each patient for determining the median value in the receiver operating characteristic (ROC) curve, which is used to select “high-risk” and “low-risk” groups. We combined clinical variables and performed univariate and multivariate Cox regression analyses in training and testing cohorts, respectively. Finally, based on the coefficients of the above multivariate Cox regression, we used “regplot” in R software to build nomogram.

### Immune Infiltration and GSEA Analysis

In the exploration of differences in immune cell infiltration, we simultaneously used 6 algorithms (TIMER, CIBERSORT, QUANTISEQ, MCP-counter, XCELL, and EPIC) to estimate the abundances of immune cells in different risk groups distinguished by risk groups. Moreover, we used the single-sample gene-set enrichment analysis (ssGSEA) and ESTIMATE algorithm to quantify immune functions and immune pathways between the low- and high-groups. More importantly, we also explored the expression levels of immune checkpoint and human leukocyte antigen related genes in different risk groups. Thereafter, gene set enrichment analysis (GSEA) was performed with differentially risk groups using “ggplot2” and “clusterProfiler” packages in R software.

### Somatic Mutation Analysis

The TCGA database was used to procure information on somatic mutations in ESCC. The analysis of mutations in ESCC patients in the high-risk and low-risk groups was performed using the “maftools” package. In this study, the tumor mutation burden (TMB) score for each sample was computed as the number of mutations divided by the length of the exon (30 Mb). All KIRC samples with somatic mutations were divided into high and low-TMB groups according to the median data. To compare the differences in survival between the low and high-TMB groups, the Kaplan-Meier analysis was performed.

### Investigation of Chemotherapeutic Efficacy

The chemotherapeutic efficacy of different risk subgroups was predicted using the “pRRophetic” R package. The half-maximal inhibitory concentration (IC50) was utilized to quantify the chemotherapeutic efficacy, and lower the IC50, the higher was the chemotherapeutic efficacy.

### Tissue Samples and Quantitative Real-Time Polymerase Chain Reaction

A total of 10 tumor tissue samples and nearby normal esophageal tissue samples were obtained from ESCC patients who underwent tumor resection. All tissue samples were collected from the Thoracic Surgery Department of the Second Hospital of Hebei Medical University with the approval by the Medical Ethics Committee of the hospital. In liquid nitrogen, fresh tumor and non-tumor tissues were frozen in a snap, preserving their integrity. The extraction of RNA was carried out using the TRIzol Reagent (Invitrogen, United States). PrimeScriptTM RT reagent Kit with gDNA Eraser (Takara) was adopted to synthesize complementary DNA (cDNA) by reverse transcription reaction. The SYBR Premix Ex Taq (Takara) was adopted for the qRT-PCR analysis. The normalization of all expression data to GAPDH as an internal control was made with the 2^−ΔΔCT^ approach. Sangon Biotech (Sangon Biotech, Shanghai, China) was applied to chemically synthesize all primers. Primers for the genes are displayed in Supplementary Table S1.

### Statistical Analysis

All statistical analyses were performed using the R software (v.4.0.1). Detailed statistical methods about transcriptome data processing are covered in the above section. *p* < 0.05 was considered statistically significant.

## Results

### Landscape of LRGs

**FIGURE 1:**
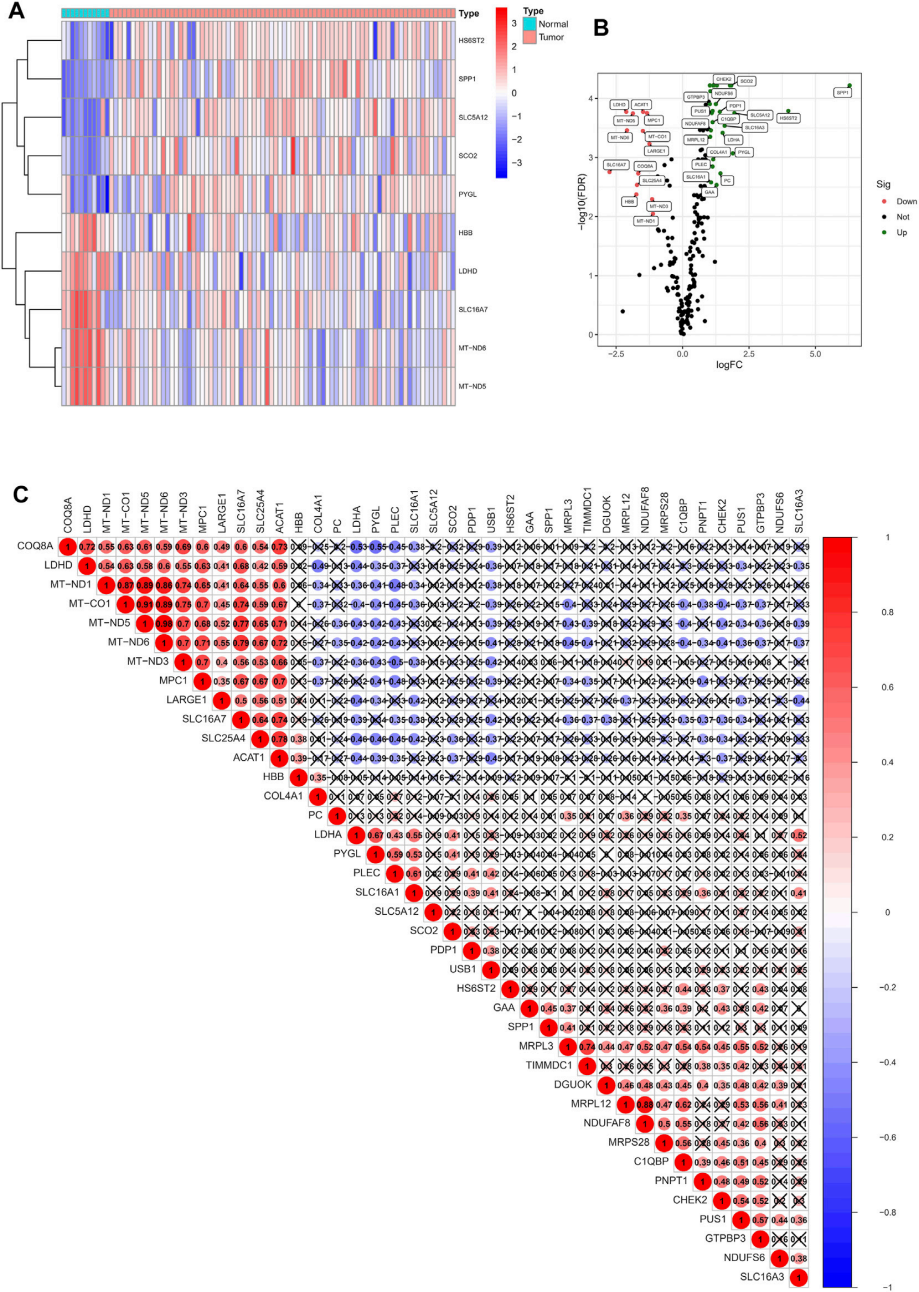
Identification of LRGs in ESCC patients. **(A)** Heatmap of the differentially expressed LRGs. **(B)** Volcano plot of differentially expressed LRGs. **(C)** The positive and negative correlation between LRGs in ESCC patients.

The flow chart of this study is shown in Supplementary Material S1. According to the expression of 262 LRGs between normal and tumor samples, we finally got 41 specific LRGs in ESCC (|Log2FC| > 1 and *p* < 0.05). The heatmap showed the TOP10 LRGs, as shown in Figure 1A. Of these, 27 were found to be up-regulated, and 14 were down-regulated (Figure 1B). As shown in Figure 1C, the correlation analysis of the 27 LRGs showed that MT-ND3 had the strongest positive correlation with MT-ND5 (r = 0.98); PYGL had the strongest negative correlation with COQ8A (r = -0.55).

### Identification of LRLs

**FIGURE 2:**
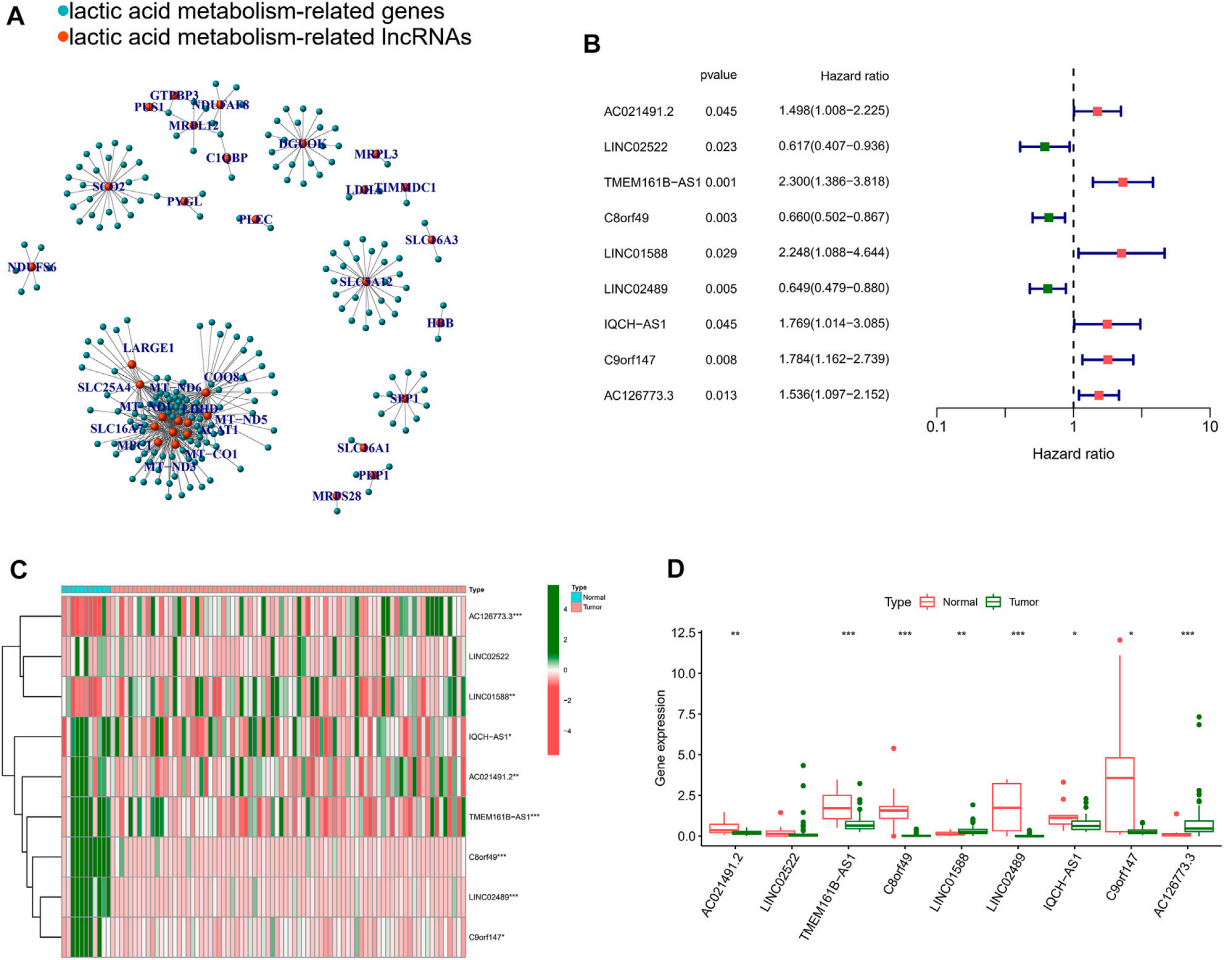
Identification of lactic acid metabolism-related prognostic lncRNAs. **(A)** The network of the 216 lactic acid metabolism-related lncRNAs. **(B)** Forrest plot of 9 lactic acid metabolism-related lncRNAs related with prognosis. **(C)** The heatmap of the 9 lactic acid metabolism-related lncRNAs. **(D)** Differential lncRNAs level between tumor tissues and normal tissues. *p < 0.05, **p < 0.01, ***p < 0.001.

For TCGA-ESCC cohort, Pearson correlation analysis was performed using the above 27 NRGs and all annotated lncRNAs (correlation coefficients >0.6 and *p* < 0.001). 216 LRLs were we identified (Figure 2A). To identify survival-related LRLs, univariate Cox analyses were performed. Finally, 9 LRLs were screened for subsequent analyses (Figure 2B). Interestingly, the Wilcox test revealed that other survival-related LRLs were highly expressed in the normal samples except AC126773.3, LINC01588, which was highly expressed in the tumor samples (Figures 2C,D).

### Construction of A Risk Signature

**FIGURE 3:**
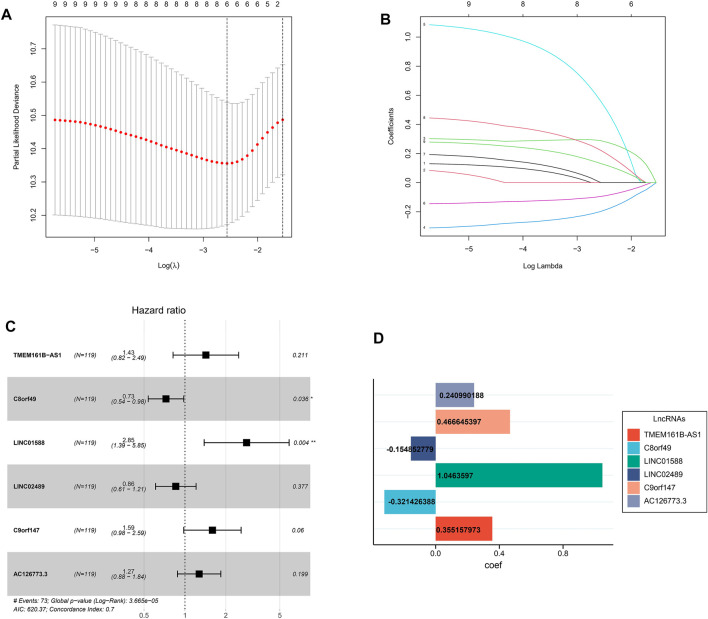
Construction of a prognostic model. **(A)** Cross-validation for tuning parameter selection in the proportional hazards model. **(B)** LASSO coefficient profiling of the 9 lactic acid metabolism-related lncRNAs. **(C)** Multivariate Cox regression analysis selected 6 lactic acid metabolism-related lncRNAs correlated with survival. **(D)** Multivariate cox regression analysis showed coefficients of each lactic acid metabolism-related lncRNAs.

For further decreasing the number of genes in the signature, the subjection of 9 LRLs to LASSO regression analysis was performed in GEO cohort (Figures 3A,B). Thereafter, 6 LRLs from LASSO were retrieved and subjected to multivariate Cox regression analysis to develop a risk stratification system (Figure 3C). Finally, combining the expression of 6-LRLs and regression coefficients (Figure 3D), the risk score of ESCC patients is calculated as follows: Risk score = (0.3551 * TMEM161B-AS1) + (−0.3214 *C8orf49) + (1.0463 * LINC01588) + (−0.1548 * LINC02489) + (0.4666 *C9orf147) + (0.2409 * AC126773.3).

### Clinical Benefits of Risk Signature

**FIGURE 4:**
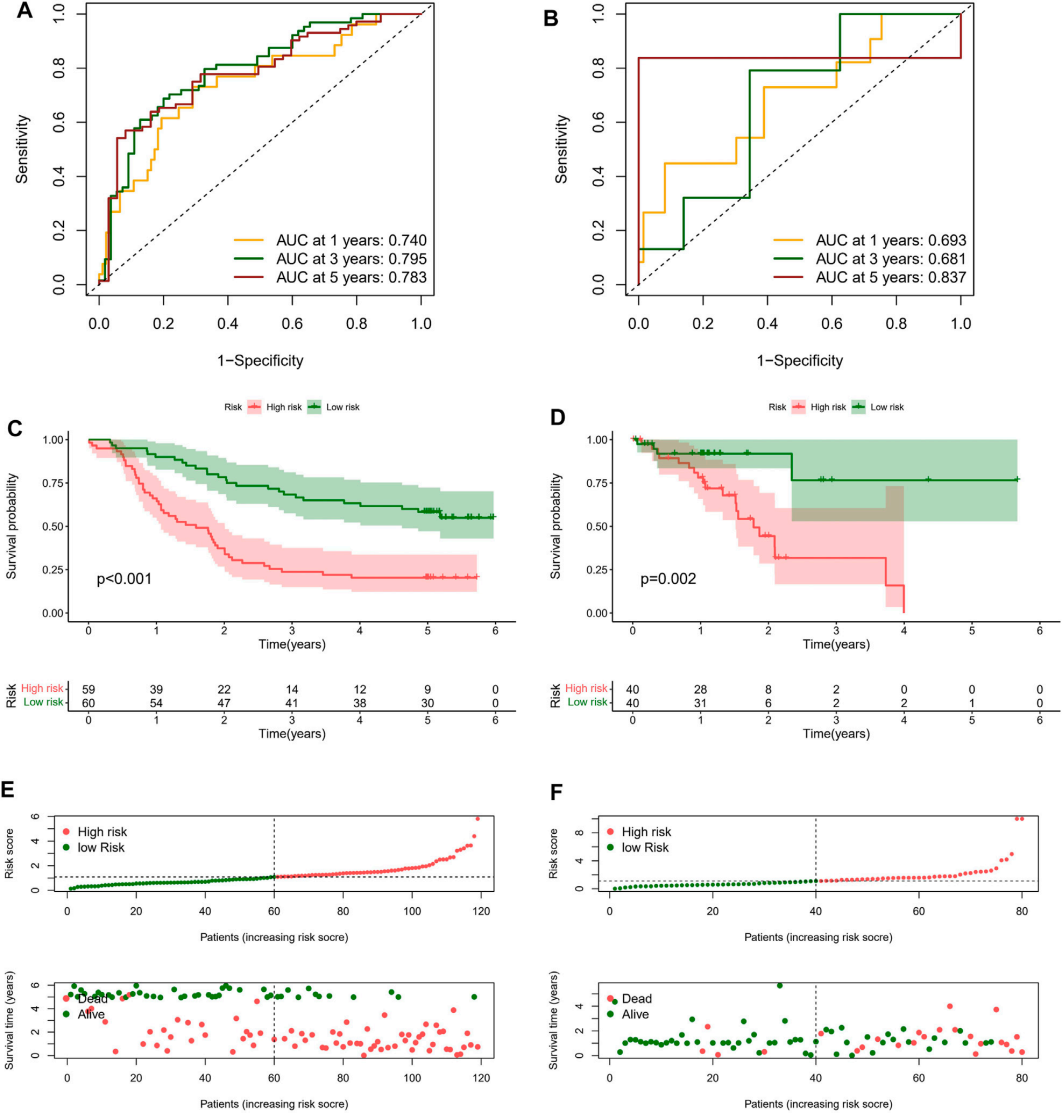
Clinical benefits of prognostic model. ROC curves and their AUC value represented 1-, 3-, and 5-years predictions in GEO **(A)** and TCGA cohorts **(B)**. Kaplan-Meier curves showed that the high-risk group had worse survival probability than the low-risk group in GEO **(C)** and TCGA cohorts **(D)**. **(E,F)** Risk score distribution plot showed the distribution of high-risk and low-risk ESCC patients; Scatter plot showed the correlation between the survival status and risk score of ESCC patients.

On basis of the median value of risk scores, ESCC patients from the GEO cohort were divided into two risk subgroups: high-risk and low-risk. As shown in Figure 4A, the AUCs of risk score performed with GEO cohort were 0.740, 0.795, and 0.783 at 1, 3, and 5 years. Meanwhile he AUC for predicting 1, 3, and 5 years were 0.693, 0.681, and 0.837 in TCGA cohort (Figure 4B). In each group, the survival time of high-risk patients was significantly shorter than that low-risk population (*p* < 0.05), as shown in Figures 4C,D. In addition, with the risk score formula, the distribution of risk score between low- and high-risk groups in GEO and TCGA cohorts. These all indicated the high-risk group had worse prognoses (Figures 4E,F). To better assess the prognostic ability of the risk signature, we performed a stratification analysis to confirm whether it retains its ability to predict OS in various subgroups. The results showed that the high-risk subgroup had worse OS compared to the low-risk subgroup in different TNM stages (*p* < 0.05; Supplementary Figure S2). These data indicated that it could be a potential predictor for ESCC patients.

**FIGURE 5:**
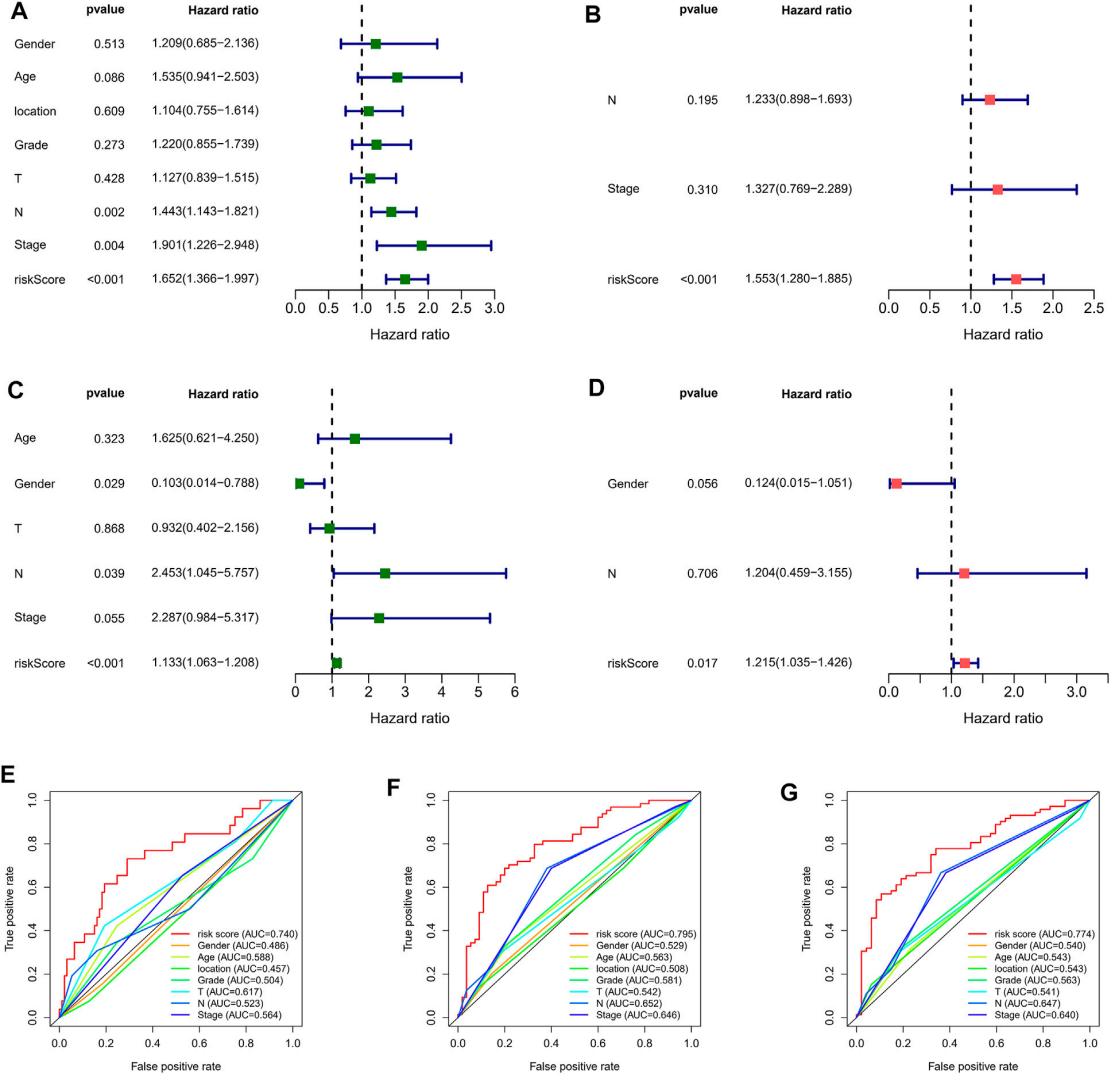
Independent prognostic value of risk score. Univariate Cox regression analysis of the risk scores in GEO **(A)** and TCGA cohorts **(B)**. Multivariate Cox regression analysis of the risk scores in GEO **(C)** and TCGA cohorts **(D)**. ROC curves of 1- **(E)**, 3- **(F)**, and 5-years **(G)** OS predicted by the nomogram.

For determining whether the risk score was an independent prognostic element for ESCC patients, univariate and multivariate Cox regression analyses were conducted using the clinical features and risk score. Based on the outcomes of univariate Cox regression analysis, the risk score was greatly related to OS in both GEO and TCGA cohorts (GEO cohort: HR = 1.652, 95% CI = 1.366–1.997; TCGA cohort: HR = 1.113, 95% CI 1.063–1.208) (Figures 5A–C). After the adjusting other confounders, the risk score was still an independent predictor of OS in multivariate Cox regression analysis (GEO cohort: HR = 1.563, 95% CI = 1.280–1.885; TCGA cohort: HR = 1.215, 95% CI = 1.035–1.426), as shown in Figures 5B–D. In addition, ROC curve analysis revealed that the risk score showed better predictive performance than the other clinicopathological features in 1-year (Figure 5E), 3-years (Figure 5F), and 5-years (Figure 5G). Moreover, The results were also exciting: in the C-index for predicting the meta cohort, the risk signature of our study showed better predictive value than the immune-related lncRNA signature established by Zhu, autophagy-related lncRNA signature established by Shi, and the metabolic syndrome-related signature established by Liu, as shown in Supplementary Figure S2.

### Construction of Nomogram

**FIGURE 6:**
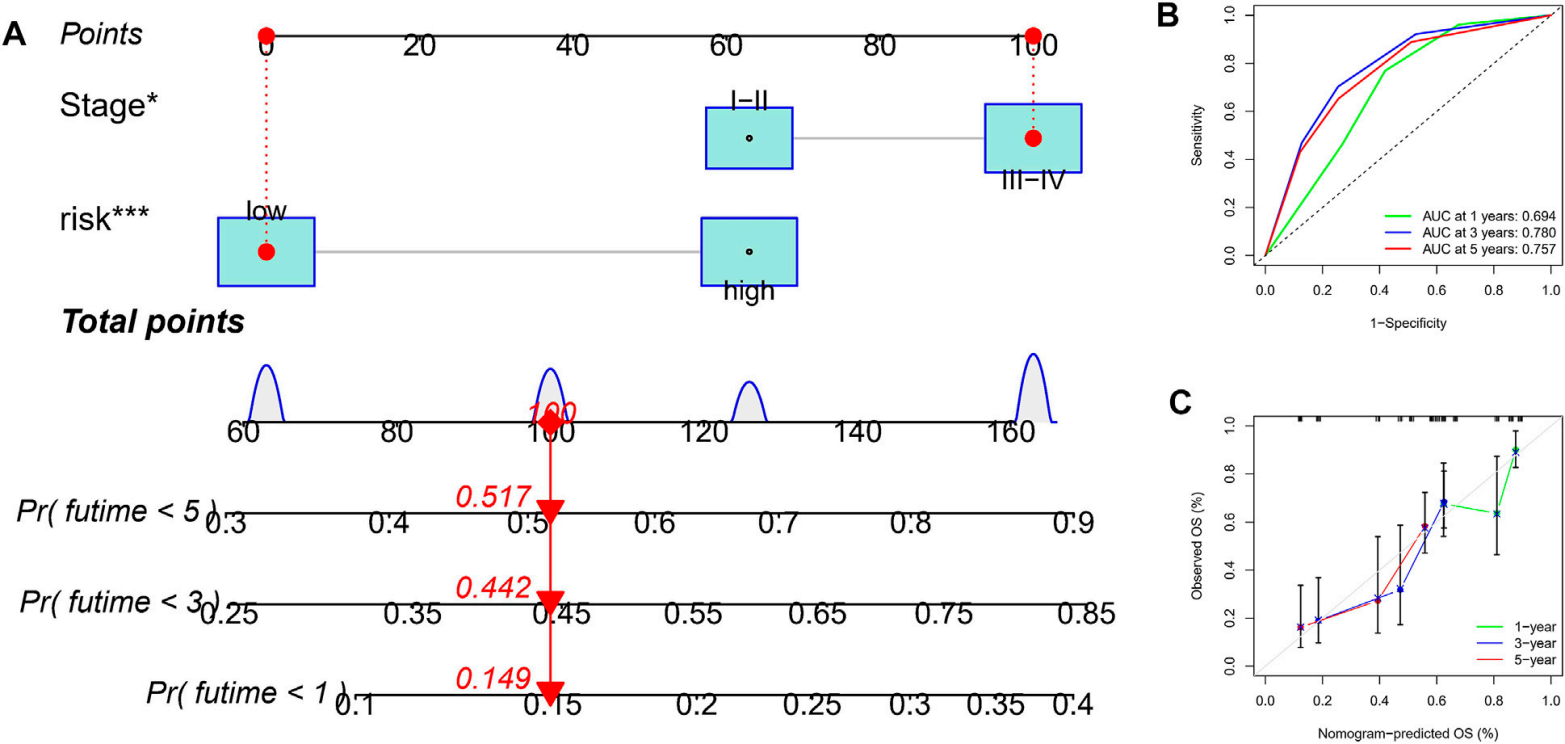
Construction and verification of nomogram. **(A)** A nomogram based on TNM stage and risk score. **(B)** ROC curves of the nomogram for OS prediction at 1, 3, and 5 years. **(C)** Calibration curves of nomogram for OS prediction at 1, 3, and 5 years.

Considering that the formula of the risk signature is complicated, the nomogram can intuitively apply to clinical work, so we visualized the risk signature based on the above risk formula. We combined significant indicators in clinical work to construct a visual prognostic signature (C-index = 0.794 [95%CI = 0.763–0.846]), as shown in Figure 6A. Moreover, the calibration curve of the nomogram showed that the prediction curves are close to the standard curve in ESCC patients, which indicates that the predicted survival rate is closely related to the actual rates at 1, 3, and 5 years, as shown in Figure 6B. Furthermore, according to the ROC analysis, good predictive performance in nomogram were revealed (Figure 6C).

### Immunity And Pathway Enrichment Analysis

**FIGURE 7:**
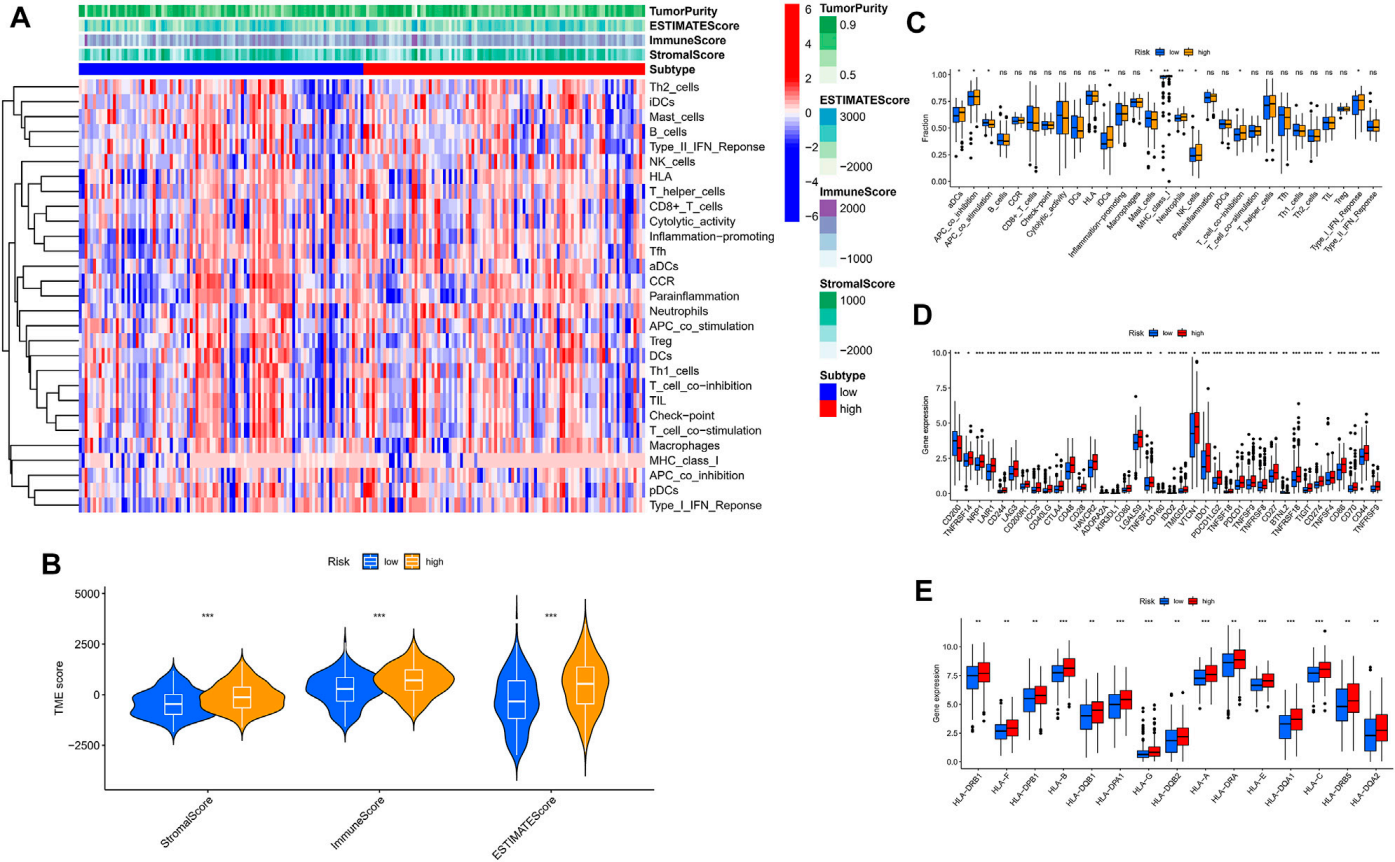
The low- and high-risk groups display different immune statuses. **(A)** Heatmap of the distribution of 29 immune cells between the low- and high-risk groups using ssGSEA. **(B)** Expression level of estimate score, immune score, and stromal score between the low- and high-risk groups. **(C)** Distinction of enrichment of immune-related signatures between low- and high-risk groups. **(D)** Comparison of 38 immune checkpoint blockade-related genes expression levels in two risk score subgroups. **(E)** Comparison of 15 HLA-related genes expression levels in two risk score subgroups. *p < 0.05, **p < 0.01, ***p < 0.001.

**FIGURE 8:**
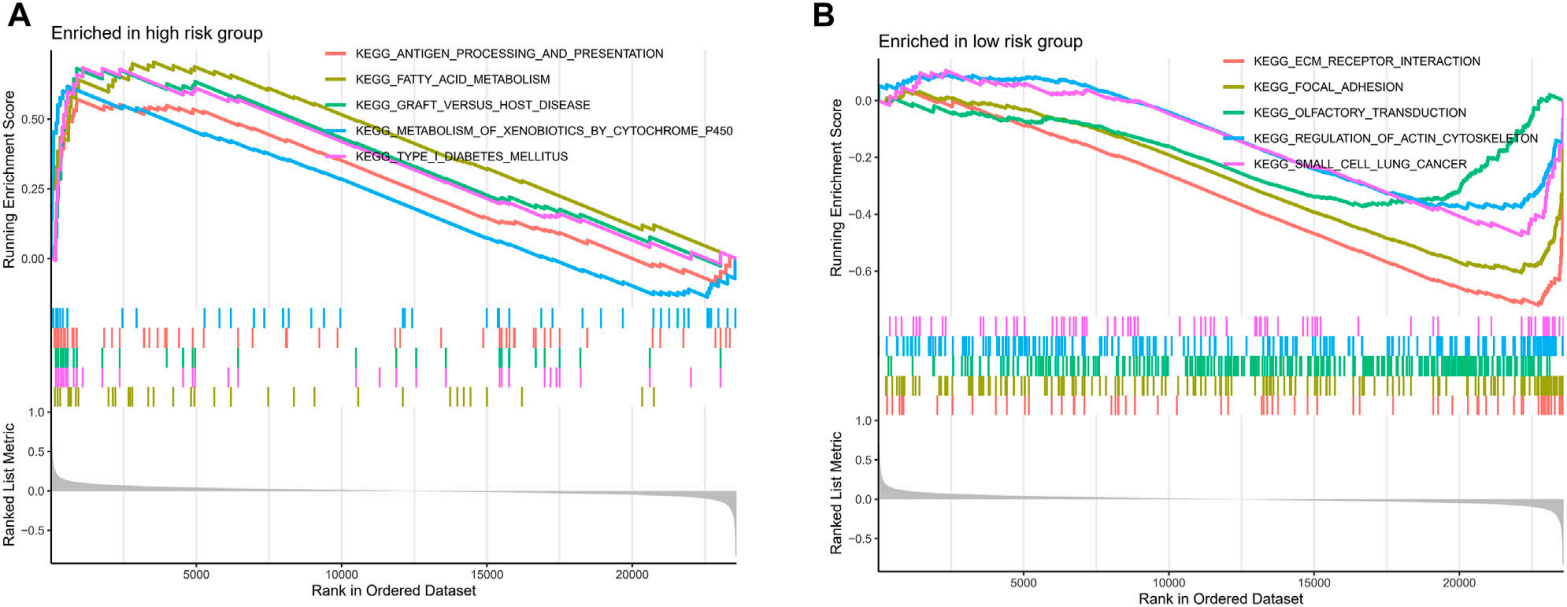
The GSEA analysis of the high- and low-risk groups. The GSEA pathway enrichment analysis of the high- **(A)** and low-risk **(B)** groups.

For further exploring the associations between the risk scores and immune cells and functions in all cohorts, the RNA-seq data from TCGA and GEO cohorts were batched using “sva” package. The enrichment scores of immune cell subpopulations and their associated functions were quantified with the ssGSEA package (Figure 7A). Meanwhile, results of ESTIMATE algorithm revealed high-risk group had a higher score in stromal, immune, and estimate (Figure 7B). Wilcox test of immunologic function confirmed significant differences between low- and high-risk groups for other immunological function, as shown in Figure 7C. These results suggested that the high-risk group may be more prone to respond in immunotherapy. Moreover, it is worth noting that given the importance of checkpoint immunotherapy and human leukocyte antigen, with significant differences in the expression of immune checkpoints and HLA between different risk groups (Figures 7D,E). Finally, GSEA algorithm was used to explore the main enrichment pathways of different risk groups. In the high-risk group, FATTY ACID METABOLISM, ANTIGEN PROCESSING AND PRESENTATION and GRAFT VERSUS HOST disease were dominant (Figure 8A). In the low-risk group, important pathways were ECM RECEPTOR INTERACTION, FOCAL ADHESION, and OLFACTORY TRANSDUCTION (Figure 8B).

### Tumor Somatic Mutational Landscape

**FIGURE 9:**
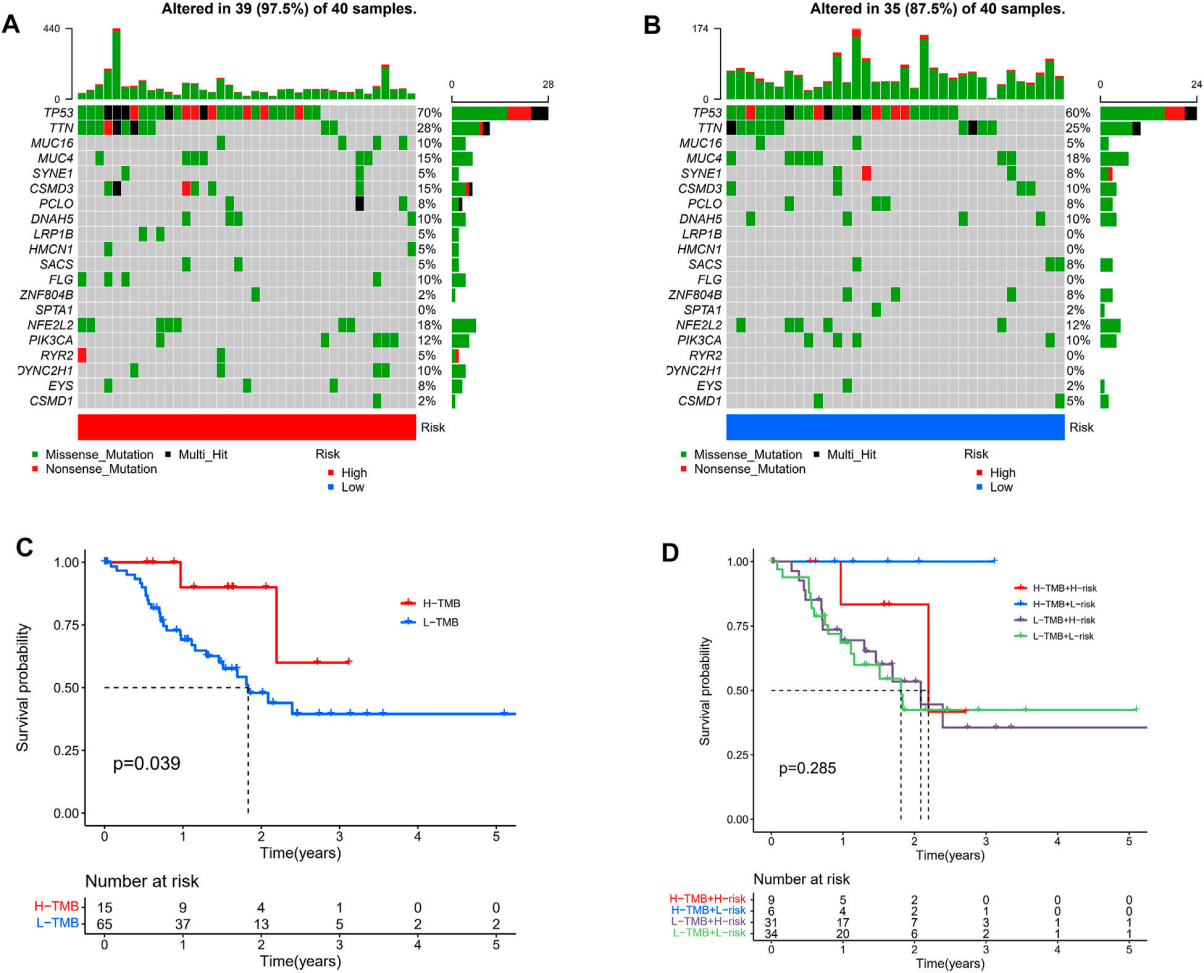
Tumor somatic mutational analyses between high- and low-risk scores. Waterfall plot shows the mutation distribution of the top 20 most frequently mutated genes in the high-risk group **(A)** and low-risk group **(B)**. **(C)** Survival probability in ESCC patients between high- and low-TMB groups. **(D)** High TMB combined with low risk score has the best prognostic indicator significance.

Given that gene mutations are a significant contributor to carcinogenesis, we investigated the differences in the distribution of somatic mutations between the high- and low-risk populations. The top 20 most frequently mutated genes in each of these two groups are shown in Figures 9A,B. Kaplan-Meier curves for OS showed that the patients in the high-TMB group had better prognostic outcomes (Figure 9C). Subsequently, we combined the risk score with the TMB score and found that the prognosis of ESCC patients was the best when the low-risk and high TMB scores were combined (Figure 9D).

### Correlation of the Prognostic Signature With Chemotherapeutic Efficacy

**FIGURE 10:**
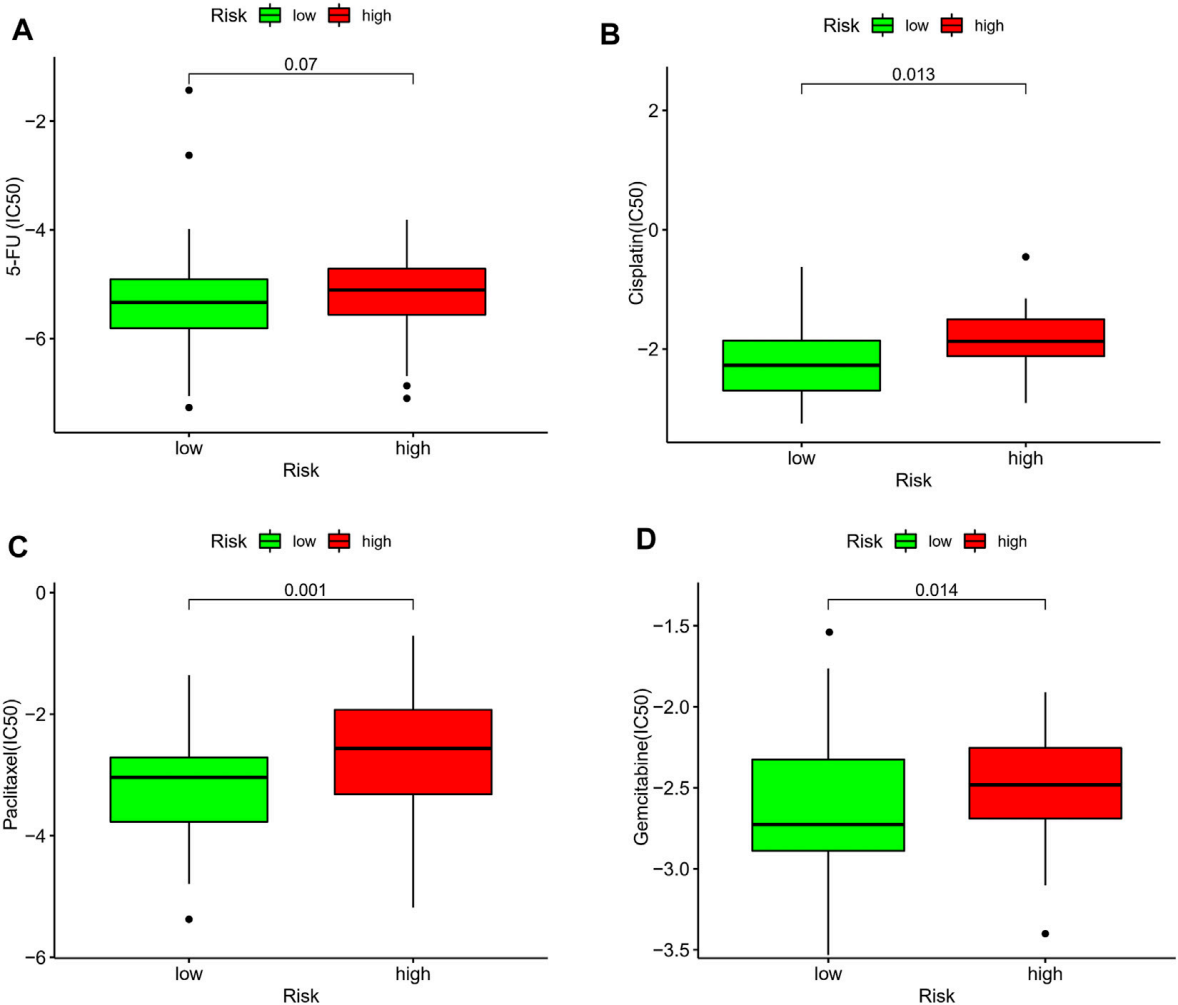
Estimation of the associations between chemosensitivity and the prognostic model. The ICI50 of **(A)** 5-fluorouracil, **(B)** cisplatin, **(C)** paclitaxel, and **(D)** gemcitabine, in the high-risk and low-risk groups.

The current first-line chemotherapy regimens for ESCC in clinical guidelines are: platinum-containing dual-agent chemotherapy regimens, paclitaxel combined with platinum regimens, or 5-fluorouracil-based regimens. We used the pRRophetic algorithm to evaluate the therapeutic effect of the drug. Unfortunately, there was no difference in the expression of 5-fluorouracil between the different risk groups (Figure 10A). In addition, the IC50 results showed that the low-risk group had better efficacy on cisplatin (Figure 10B), paclitaxel (Figure 10C), and gemcitabine (Figure 10D) than the high-risk group.

### Identification of Expression Levels of the 6 Lactic Acid Metabolism-Related LncRNAs

**FIGURE 11:**
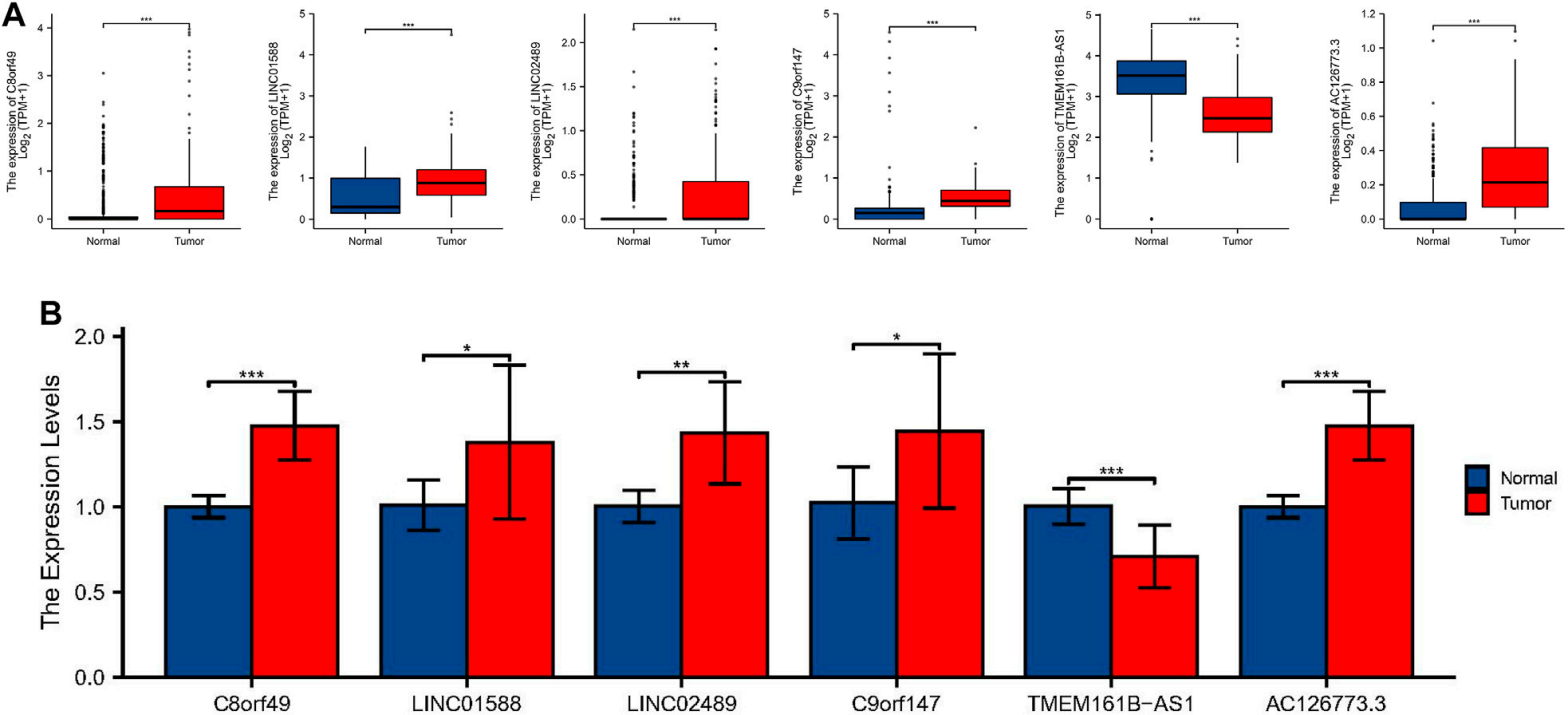
Identification of expression levels of the 6 lactic acid metabolism-related lncRNAs. **(A)** The differential expression of C8orf49, LINC01588, LINC02489, C9orf147, TMEM161B-AS1, and AC126773.3 in ESCC samples compared to adjacent normal controls in TCGA and GTEx RNA-seq data. **(B)** The expression levels of the 6 lncRNAs in 10 pairs ESCC and matched adjacent normal tissues were examined by qRT-PCR. *p < 0.05, **p < 0.01, ***p < 0.001.

To further demonstrate the feasibility of the prognostic model, we first analyzed the expression levels of 6 lncRNAs in ESCC samples and normal control samples using TCGA and GTEx RNA-seq data. The results showed that the expression level of C8orf49, LINC01588, LINC02489, C9orf147, and AC126773.3 were significantly up-regulated in ESCC samples compared with normal control samples, while TMEM161B-AS1 was down-regulated in ESCC samples (Figure 11A). In consistence with the RNA-seq data, qRT-PCR analysis of clinical tissue samples also showed that the expression of C8orf49, LINC01588, LINC02489, C9orf147, and AC126773.3 was increased and the expression of TMEM161B-AS1 decreased in ESCC tissues compared with adjacent normal esophageal tissues (Figure 11B). Collectively, these findings further validated the stability and reliability of the LRLs prognostic signature.

## Discussion

To our knowledge, this is the first study to explore the association between LRLs and ESCC. The univariate Cox regression analysis in the TCGA cohort revealed that 9 LRLs were substantially associated with prognosis. LASSO regression and multivariate Cox regression analyses were used in the GEO cohort. 6-LRLs were identified as independent prognostic factors and were used to construct a prognostic risk-related signature. To evaluate the predictive ability of the risk signature, we divided the patients with ESCC into low and high-risk groups based on the median value of the risk scores. Subsequently, we conducted the Kaplan-Meier survival analysis, which revealed that the high-risk group had a lower survival probability than the low-risk group in both the GEO and TCGA cohorts. In multivariate analysis, the risk signature was shown to be an independent prognostic factor, and it was found to be a better predictor of the prognosis for ESCC than the currently used grading and staging approaches. The established nomogram is suitable for clinical applications to predict the 1-, 3-, and 5-years survival rates of patients. A significant correlation was observed between the lncRNA-based prognostic signature and immune-cell infiltration, TME, tumor somatic mutations, and chemotherapeutic drug sensitivity. Finally, we used GTEx RNA-seq data and qRT-PCR experiments to verify the expression levels of 6 LRLs. In conclusion, we constructed a prognostic signature based on 6 LRLs that could predict the prognosis and immunotherapy response of patients with ESCC.

Among these 6 signature-related lncRNAs, the expression of TMEM161B-AS1 was significantly decreased in ESCC patients. TMEM161B-AS1gene is located on chromosome 5q14.3. A recent study showed that overexpression of TMEM161B-AS1 inhibited proliferation, invasion, and glycolysis, as well as glucose uptake and lactic acid generation in ESCC cells (Shi et al., 2021). However, the involvement and function of the remaining five model-related lncRNAs (C8orf49, LINC01588, LINC02489, C9orf147, and AC126773.3) in other diseases or malignancies has not yet been reported. These genes were, therefore, considered as promising biomarkers that need further investigation.

According to the previous studies, an increasing number of studies focused on the TME have highlighted the importance of lncRNAs in immune infiltration. The lncRNA MIAT is substantially linked with immune cell infiltration and may play a critical role in the immune escape of HCC (Peng et al., 2020). Researchers from Huang Di et al. (Huang et al., 2018) discovered that NKILA lncRNA enhances tumor immune evasion by increasing the susceptibility of T lymphocytes to activation-induced cell death. We found that the lncRNA-based prognostic signature was substantially linked to immune cell infiltration in the current study. In the ESTIMATE study, the risk score was favorably connected with both the estimate, stromal, and immune scores, suggesting that the prognostic signature could be used as a new immunological indicator in the treatment of ESCC. Furthermore, the ssGSEA analysis revealed that infiltrating immune cells (i.e., aDCs, iDCs, neutrophils, mast cells, and NK cells) were significantly increased, and immune signatures (i.e., APC coinhibition, APC costimulation, T cell coinhibition, IFN response type I, and MHC class I) were remarkably activated when the risk score was elevated. The expression of 38 immune check blockade-related genes and 15 HLA-related genes increased considerably with increasing risk scores, indicating that samples with low-risk scores may respond to immunotherapy. Taken together, these findings indicate that the prognostic signature may aid in the discovery of the regulatory mechanism of tumor immunity and provide new insights for future TME investigations.

Chemotherapy remains the first-line treatment for advanced and metastatic ESCC. Owing to tumor heterogeneity in ESCC, the ESCC cases display variable sensitivity to chemotherapy (Blum Murphy et al., 2016). Therefore, we assessed the predictive value of the prognostic signature for chemo-sensitivity in patients with ESCC. According to the IC50 values, cisplatin, paclitaxel, and gemcitabine showed a better response when used in the treatment of ESCC patients with a low-risk score. Therefore, our prognostic signature may function as a promising predictor of chemotherapeutic efficacy and may help in identifying the most appropriate chemotherapy regimen for each with ESCC.

To the best of our knowledge, this study is the first to construct a prognostic 6-LRL-based risk signature validated in patients with ESCC. Functional enrichment, immune cell infiltration, tumor somatic mutations and chemosensitivity were also analyzed. Despite its many advantages, the current study has some limitations. First, prospective, larger, multicenter trials are warranted to provide more significant evidence for clinical applications. Furthermore, as the lncRNAs screened by the study have not been previously analyzed, further research into their regulatory mechanisms is required.

In conclusion, the 6-lncRNA signature constructed in our study is a reliable and robust marker to predict the survival outcome of ESCC patients. The signature was robustly associated with immune infiltration levels, TMB scores, and chemo-sensitivity. In addition, we find that the risk score–based stratification could guide personal chemotherapy and immunotherapy for individual tumors, improving the prognosis of ESCC patients.

## Data Availability

The datasets presented in this study can be found in online repositories. The names of the repository/repositories and accession number(s) can be found in the article/Supplementary Material.

## Supplementary Material

Supplementary Figure S1 Survival analysis in different TNM stage. **(A)** Survival analysis of I-II staging in TCGA cohort. **(B)** Survival analysis of III-IV staging in TCGA cohort. **(C)** Survival analysis of I-II staging in GEO cohort. **(D)** Survival analysis of III-IV staging in GEO cohort.

Supplementary Figure S2 C-index of different signatures.

## Abbreviations

lncRNAs, long noncoding RNAs; ESCC, esophageal squamous cell carcinoma; EAC, esophageal adenocarcinoma; GEO, gene expression omnibus; TCGA, the cancer genome atlas; LRGs, lactic acid metabolism-related genes; DEGs, differentially expressed genes; LASSO, least absolute shrinkage and selection operator; ROC, receiver operating characteristic; AUC, area under the curve; OS, overall survival; GSVA, gene set enrichment analysis; ssGSEA, single-sample gene-set enrichment analysis; TME, tumor microenvironment; TMB, tumor mutation burden; IC50, half maximal inhibitory concentration; qRT-PCR, quantitative real-time polymerase chain reaction; HLA, human leukocyte antigen.

## References

[B1] Abbaszadeh Z. Çeşmeli S. Biray Avcı Ç. (2020). Crucial Players in Glycolysis: Cancer Progress. Gene 726, 144158. 10.1016/j.gene.2019.144158 31629815

[B2] Abdel-Wahab A. F. Mahmoud W. Al-Harizy R. M. (2019). Targeting Glucose Metabolism to Suppress Cancer Progression: Prospective of Anti-glycolytic Cancer Therapy. Pharmacol. Res. 150, 104511. 10.1016/j.phrs.2019.104511 31678210

[B3] Abnet C. C. Arnold M. Wei W.-Q. (2018). Epidemiology of Esophageal Squamous Cell Carcinoma. Gastroenterology 154, 360–373. 10.1053/j.gastro.2017.08.023 28823862 PMC5836473

[B4] Anandavadivelan P. Lagergren P. (2016). Cachexia in Patients with Oesophageal Cancer. Nat. Rev. Clin. Oncol. 13, 185–198. 10.1038/nrclinonc.2015.200 26573424

[B5] Bai Y. Lin H. Chen J. Wu Y. Yu S. a. (2021). Identification of Prognostic Glycolysis-Related lncRNA Signature in Tumor Immune Microenvironment of Hepatocellular Carcinoma. Front. Mol. Biosci. 8, 645084. 10.3389/fmolb.2021.645084 33968985 PMC8100457

[B6] Blum Murphy M. A. Elimova E. Ajani J. A. (2016). Current Concepts and Future Potential in Neoadjuvant Chemotherapy for Esophageal Cancer. Expert Rev. Gastroenterol. Hepatol. 10 (3), 383–392. 10.1586/17474124.2016.1116936 26560689

[B7] Faubert B. Li K. Y. Cai L. Hensley C. T. Kim J. Zacharias L. G. (2017). Lactate Metabolism in Human Lung Tumors. Cell 171 (2), 358–371. e9. 10.1016/j.cell.2017.09.019 28985563 PMC5684706

[B8] He Y. Liang D. Du L. Guo T. Liu Y. Sun X. (2020). Clinical Characteristics and Survival of 5283 Esophageal Cancer Patients: A Multicenter Study from Eighteen Hospitals across Six Regions in China. Cancer Commun. 40, 531–544. 10.1002/cac2.12087 PMC7571391 32845581

[B9] Hirschhaeuser F. Sattler U. G. A. Mueller-Klieser W. (2011). Lactate: A Metabolic Key Player in Cancer: Figure 1. Cancer Res. 71 (22), 6921–6925. 10.1158/0008-5472.CAN-11-1457 22084445

[B10] Huang D. Chen J. Yang L. Ouyang Q. Li J. Lao L. (2018). NKILA lncRNA Promotes Tumor Immune Evasion by Sensitizing T Cells to Activation-Induced Cell Death. Nat. Immunol. 19 (10), 1112–1125. 10.1038/s41590-018-0207-y 30224822

[B11] Li Z. Qin X. Bian W. Li Y. Shan B. Yao Z. (2019). Exosomal lncRNA ZFAS1 Regulates Esophageal Squamous Cell Carcinoma Cell Proliferation, Invasion, Migration and Apoptosis via microRNA-124/STAT3 axis. J. Exp. Clin. Cancer Res. 38 (1), 477. 10.1186/s13046-019-1473-8 31775815 PMC6882153

[B12] Peng L. Chen Y. Ou Q. Wang X. Tang N. (2020). LncRNA MIAT Correlates with Immune Infiltrates and Drug Reactions in Hepatocellular Carcinoma. Int. Immunopharmacology 89 (Pt A), 107071. 10.1016/j.intimp.2020.107071 33221703

[B13] Peng Y. Xu C. Wen J. Zhang Y. Wang M. Liu X. (2021). Fatty Acid Metabolism-Related lncRNAs Are Potential Biomarkers for Predicting the Overall Survival of Patients with Colorectal Cancer. Front. Oncol. 11, 704038. 10.3389/fonc.2021.704038 34458145 PMC8386021

[B14] Pereira-Nunes A. Afonso J. Granja S. Baltazar F. (2020). Lactate and Lactate Transporters as Key Players in the Maintenance of the Warburg Effect. Adv. Exp. Med. Biol. 1219, 51–74. 10.1007/978-3-030-34025-4_3 32130693

[B15] Shi Z. Li G. Li Z. Liu J. Tang Y. (2021). TMEM161B‐AS1 Suppresses Proliferation, Invasion and Glycolysis by Targeting miR‐23a‐3p/HIF1AN Signal axis in Oesophageal Squamous Cell Carcinoma. J. Cell Mol Med 25 (14), 6535–6549. 10.1111/jcmm.16652 34046994 PMC8278070

[B16] Smyth E. C. Lagergren J. Fitzgerald R. C. Lordick F. Shah M. A. Lagergren P. (2017). Oesophageal Cancer. Nat. Rev. Dis. Primers 3, 17048. 10.1038/nrdp.2017.48 28748917 PMC6168059

[B17] Xiao Z.-D. Zhuang L. Gan B. (2016). Long Non-coding RNAs in Cancer Metabolism. Bioessays 38 (10), 991–996. 10.1002/bies.201600110 27550823 PMC5144540

[B18] Yang J. Liu X. Cao S. Dong X. Rao S. Cai K. (2020). Understanding Esophageal Cancer: The Challenges and Opportunities for the Next Decade. Front. Oncol. 10, 1727. 10.3389/fonc.2020.01727 33014854 PMC7511760

[B19] Yoshida G. J. (2015). Metabolic Reprogramming: the Emerging Concept and Associated Therapeutic Strategies. J. Exp. Clin. Cancer Res. 34, 111. 10.1186/s13046-015-0221-y 26445347 PMC4595070

[B20] Zhao Q. Fan C. (2019). A Novel Risk Score System for Assessment of Ovarian Cancer Based on Co-expression Network Analysis and Expression Level of Five lncRNAs. BMC Med. Genet. 20 (1), 103. 10.1186/s12881-019-0832-9 31182053 PMC6558878

[B21] Zheng J. Guo J. Zhang H. Cao B. Xu G. Zhang Z. (2021). Four Prognosis-Associated lncRNAs Serve as Biomarkers in Ovarian Cancer. Front. Genet. 12, 672674. 10.3389/fgene.2021.672674 34367239 PMC8336869

[B22] Zhou M. Sun Y. Sun Y. Xu W. Zhang Z. Zhao H. (2016). Comprehensive Analysis of lncRNA Expression Profiles Reveals a Novel lncRNA Signature to Discriminate Nonequivalent Outcomes in Patients with Ovarian Cancer. Oncotarget 7 (22), 32433–32448. 10.18632/oncotarget.8653 27074572 PMC5078024

[B23] Zhou M. Wang X. Shi H. Cheng L. Wang Z. Zhao H. (2016). Characterization of Long Non-coding RNA-Associated ceRNA Network to Reveal Potential Prognostic lncRNA Biomarkers in Human Ovarian Cancer. Oncotarget 7 (11), 12598–12611. 10.18632/oncotarget.7181 26863568 PMC4914307

