# Supplementary figures and images for: Identification of A Risk Signature Based on Lactic Acid Metabolism-Related LncRNAs in Patients With Esophageal Squamous Cell Carcinoma
Source: Front Cell Dev Biol. 2022 May 12;10:845293. doi: 10.3389/fcell.2022.845293 (PMC9134121; doi:10.3389/fcell.2022.845293)

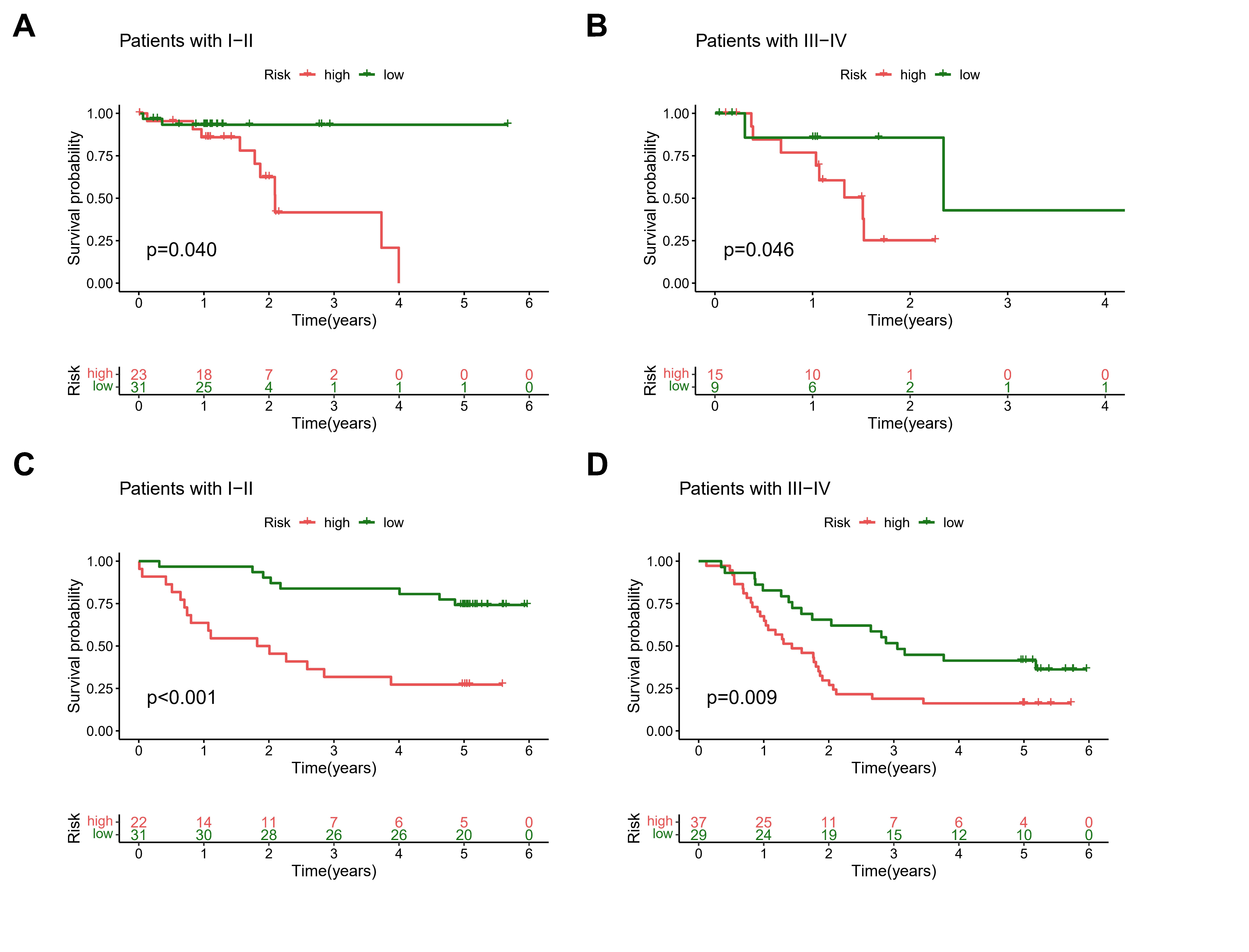

Supplement: Supplementary file 1 [file Image1.TIFF]

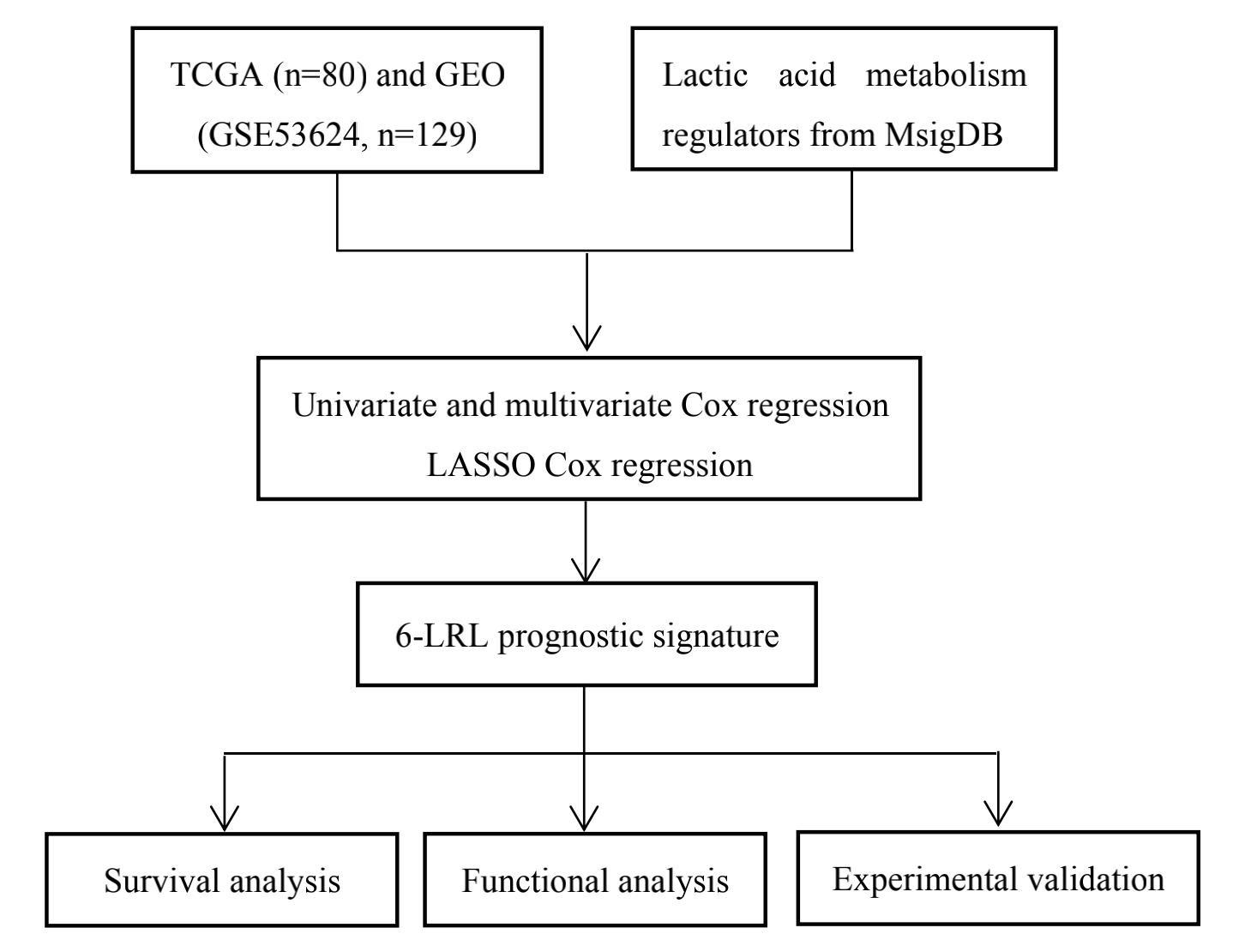


**The flow chart of this study.**

Supplement: Supplementary file 3 [file Table2.DOCX]

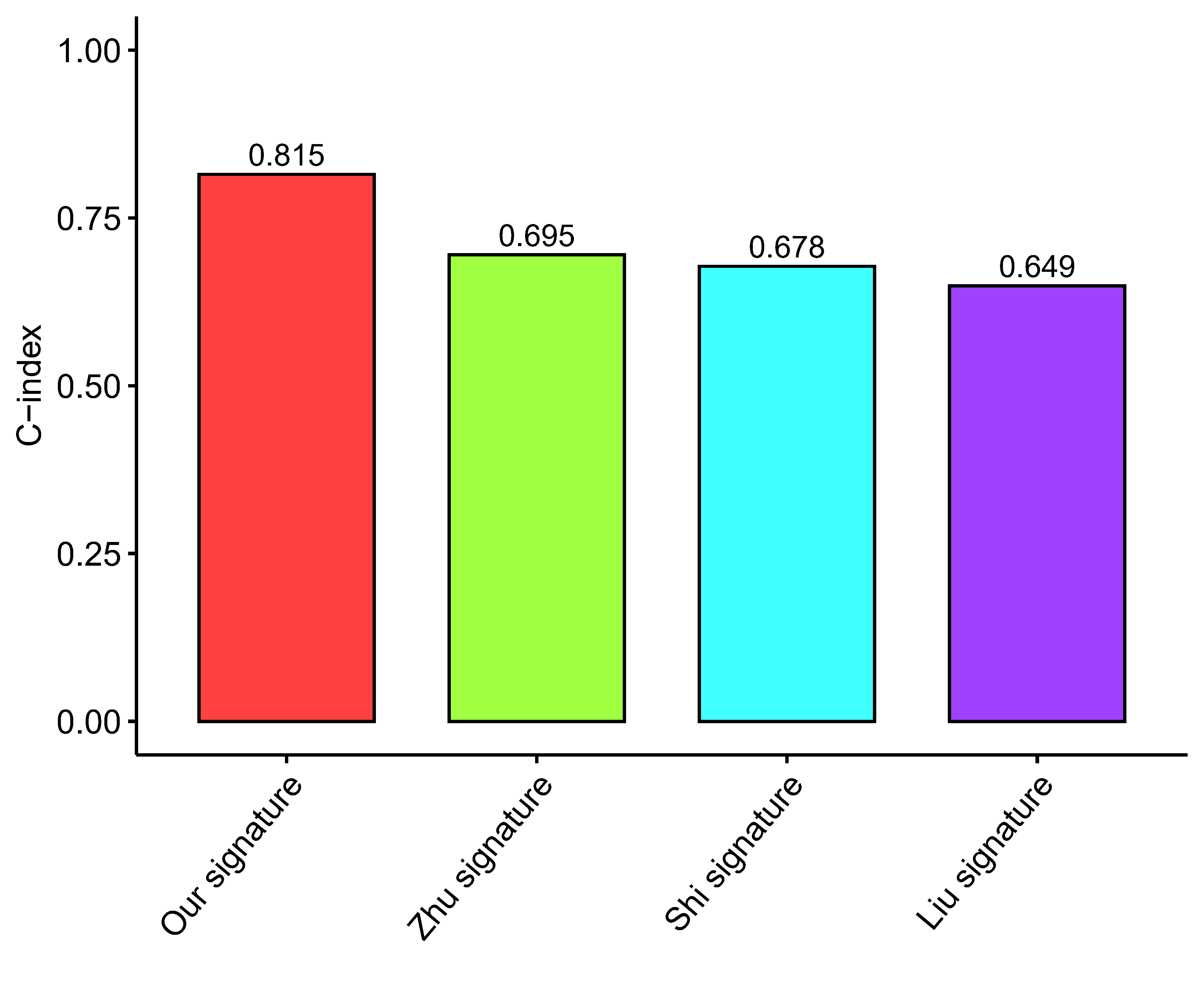

Supplement: Supplementary file 4 [file Image2.TIFF]
