# Supplementary material for: Identification of A Risk Signature Based on Lactic Acid Metabolism-Related LncRNAs in Patients With Esophageal Squamous Cell Carcinoma
Source: Front Cell Dev Biol. 2022 May 12;10:845293. doi: 10.3389/fcell.2022.845293 (PMC9134121; doi:10.3389/fcell.2022.845293)
Supplement: Supplementary file 2 [file Table1.DOCX]

**Table S1:** The primer sequences used in the present study.

| lncRNA | Forward primer (5′-3′) | Reverse primer (5′-3′) |
| --- | --- | --- |
| C8orf49 | 5'‐TGCAGTGGTTATAGCGATCCT‐3' | 5'‐CCTTGTCTTCGCACTCTTATCC‐3' |
| LINC01588 | 5'‐TTCGCCGACGTAGCCATAGA‐3' | 5'‐CGTCCCAGTACAGGTCCCT‐3' |
| LINC02489 | 5'‐AGACTCCGCATCGCAAAGG‐3' | 5'‐TCACCACGTTGTTGTCAAGGG‐3' |
| C9orf147 | 5'‐ACTACACCGAGGAAATGGGCT‐3' | 5'‐CCCACAATGCCAGTTAAGAAGA‐3' |
| TMEM161B-AS1 | 5'‐GCAACCGCCATTCTGCTAC‐3' | 5'‐CTCCCCTATGAGCACCACTC‐3' |
| AC126773.3 | 5'‐GCTCCGGTTTTGGGGTATCTG‐3' | 5'‐GCGTTGATGTGAGGTTCCAG‐3' |
| GAPDH | 5'‐ACAACTTTGGTATCGTGGAAGG‐3' | 5'‐ACAACTTTGGTATCGTGGAAGG‐3' |
